# Ponicidin Inhibits NLRP3 Inflammasome Activation via Alleviating Mitochondrial Damage in LPS-Primed Macrophages

**DOI:** 10.3390/antiox15101325

**Published:** 2026-10-14

**Authors:** Congyu Wu, Yuan Kang, Yu Liu, Xin Huang, Muqing Wang, Min Cai, Yuan Gao, Yun Qi

**Affiliations:** 1 State Key Laboratory of Bioactive Substance and Function of Natural Medicines, Institute of Medicinal Plant Development, Chinese Academy of Medical Sciences & Peking Union Medical College, Beijing 100193, China; s2024009003@student.pumc.edu.cn (C.W.); s2024009044@student.pumc.edu.cn (M.W.);; 2 Shenzhen Medical Academy of Research and Translation (SMART), Shenzhen 518107, China; kangyuan@smart.org.cn; 3 Westlake University-SMART Joint Program, Westlake University, Hangzhou 310030, China

**Keywords:** ponicidin, mitochondria, NLRP3 inflammasome, macrophage, mtROS, mtDNA/ox-mtDNA

## Abstract

The NLRP3 inflammasome is a cytosolic pattern-recognition complex that senses sterile and infectious insults and drives innate immune responses. Herein, we identify ponicidin (PON), a natural diterpenoid isolated from *Rabdosia rubescens*, as an inhibitor of NLRP3 inflammasome activation. In LPS-primed macrophages, PON suppressed IL-1β secretion by inhibiting caspase-1 activation and attenuated nigericin- or ATP-induced pyroptosis, as indicated by reduced LDH release and GSDMD cleavage. Meanwhile, AIM2 inflammasome-mediated IL-1β production was not affected by PON. Mechanistically, PON inhibited ASC oligomerization and speck formation, critical steps in NLRP3 inflammasome assembly. It could reduce mitochondria-derived danger signals related to NLRP3 activation, such as mtROS, mtDNA and ox-mtDNA, without affecting ion fluxes (e.g., K^+^, Ca^2+^, Cl^−^). Consistently, PON preserved the mitochondrial membrane potential and intracellular ATP levels, indicating comprehensive protection of mitochondrial function. In vivo, PON markedly reduced IL-1β levels in peritoneal lavage fluid of MSU-induced peritonitis mice and lowered serum IL-1β, TNF-α, and MCP-1 levels in endotoxemia mice. Collectively, PON alleviates mitochondrial dysfunction to prevent ASC oligomerization and NLRP3 inflammasome assembly, thereby reducing the cleavage of pro-IL-1β and GSDMD. Our findings suggest that PON may be a potential candidate for treating NLRP3 inflammasomopathies.

## 1. Introduction

Inflammasomes are intracellular multiprotein complexes that serve as critical signaling platforms in innate immunity, responding to pathogen-associated molecular patterns (PAMPs) and damage-associated molecular patterns (DAMPs) through their pattern recognition receptor (PRR) components [1]. Among the currently identified inflammasomes, the NOD-like receptor family pyrin domain-containing 3 (NLRP3) inflammasome is the most extensively studied. The NLRP3 inflammasome is composed of the sensor protein NLRP3, the adaptor protein apoptosis-associated speck-like protein containing a caspase recruitment domain (ASC), and the effector molecule pro-caspase-1 [2]. Canonical NLRP3 inflammasome activation is strictly regulated by two consecutive signals. The priming signal, typically initiated by Toll-like receptor (TLR) ligands, activates the NF-κB pathway to upregulate the expression of NLRP3 and pro-IL-1β. Subsequently, a second activation signal is provided by diverse NLRP3-activating stimuli, including extracellular ATP, nigericin, and monosodium urate (MSU) crystals. This activation process induces assembly of the NLRP3 inflammasome complex, leading to the autocatalytic activation of caspase-1, which subsequently cleaves pro-IL-1β into mature IL-1β and cleaves gasdermin D (GSDMD) to generate its active pore-forming N-terminal fragment, thereby triggering pyroptosis [3]. Aberrant NLRP3 activation underlies many inflammatory diseases (gout, type 2 diabetes, Alzheimer’s disease, atherosclerosis), making it an attractive therapeutic target [4].

Mitochondria, the metabolically active organelles with membrane structures, are tightly correlated with NLRP3 inflammasome activation. External NLRP3 activators do not directly activate the NLRP3 inflammasome but rather trigger the release of mitochondria-derived molecules, including mitochondrial reactive oxygen species (mtROS), mitochondrial DNA (mtDNA), and oxidized mitochondrial DNA (ox-mtDNA), which belong to intracellular DAMPs and promote or directly induce NLRP3 inflammasome activation [5,6]. Although the role of mitochondria during the activation of the NLRP3 inflammasome is somewhat controversial [7], increasing evidence has demonstrated that mitochondria can influence the NLRP3 inflammasome in different aspects and play an essential role in NLRP3 activation. In particular, a ‘‘two-activation signal’’ model, with one signal originating from mitochondrial dysfunction and an additional signal simultaneously needed for NLRP3 activation, recently proposed by several researchers, supports the notion that mitochondrial dysfunction is indispensable for NLRP3 activation [8]. Therefore, protecting mitochondria against both intracellular and extracellular injuries can be an interventional strategy for mitigating uncontrolled NLRP3 inflammasome activation.

Ponicidin (PON) is an ent-kaurane diterpenoid, a class of compounds with broad antitumor activities [9]. Indeed, both PON and its structural analogue oridonin have efficacy against multiple types of cancers, such as lung, liver, breast, and pancreatic cancer [10,11]. In fact, their source plant *Rabdosia rubescens* (Hemsl.) Hara (*R. rubescens*) has been documented in the Chinese Materia Medica to possess antitumor activities [12]. Beyond the anticancer effects, *R. rubescens* is also used to treat inflammatory conditions such as sore throat, tonsillitis, and respiratory infections, owing to its heat-clearing, detoxifying, and analgesic properties [13]. As a major bioactive constituent of *R. rubescens*, PON has been reported to alleviate inflammatory pain and ulcerative colitis [13,14]. Nevertheless, its effect on NLRP3 inflammasome activation remains unclear. The present study systematically investigated the effect and underlying mechanism of PON on NLRP3 inflammasome activation by using multiple well-established in vitro and in vivo models.

## 2. Materials and Methods

### 2.1. Reagents

PON (Cat^#^B20311) and digitonin (Cat^#^S18140) were purchased from Yuanye Bio-Technology Co., Ltd. (Shanghai, China). Nigericin sodium salt (Cat^#^N102401) was obtained from Shanghai Aladdin Biochemical Technology Co., Ltd. (Shanghai, China). LPS (Cat^#^L4130), ATP (Cat^#^A6419) and MSU (Cat^#^U2875) were acquired from Sigma-Aldrich (St. Louis, MO, USA). MCC950 (Cat^#^GC31644) was obtained from GLPBIO Technology (Montclair, CA, USA). Opti-MEM Reduced Serum Medium (Cat^#^sc-108062) was purchased from Santa Cruz Biotechnology (Santa Cruz, CA, USA). Poly (dA:dT) naked (Cat^#^tlrl-patn) was purchased from InvivoGen (San Diego, CA, USA). Pam3CSK4 (TFA) (Cat^#^HY-P1180A) was obtained from MedChemExpress (Monmouth Junction, NJ, USA). 4-Sulfocalix[6]arene (Cat^#^S0470) was purchased from Tokyo Chemical Industry (Tokyo, Japan). Mouse IL-1β (Cat^#^432604), TNF-α (Cat^#^430901), and MCP-1 ELISA kits (Cat^#^432701) were from BioLegend (San Diego, CA, USA). MitoSOX™ red mitochondrial superoxide indicator (Cat^#^M36008) was from Thermo Fisher Scientific (Waltham, MA, USA). Enhanced ATP Assay Kit (Cat^#^S0027), Enhanced mitochondrial membrane potential assay kit with JC-1 (Cat^#^C2003S), CHAPS (Cat^#^ST1146), Hoechst 33342 dye (Cat^#^C1028), Alexa Fluor 488-labeled goat anti-rabbit IgG (H + L) (Cat^#^A0423), antifade mounting medium (Cat^#^P1028) and Vinculin rabbit Ab (Cat^#^AG3539) were obtained from Beyotime Institute of Biotechnology (Haimen, China). The antibody against GSDMD (Cat^#^Ab209845) was from Abcam (Cambridge, UK). Antibodies against NLRP3, caspase-1 and ASC (Cat^#^AG-20B-0014-C100, AG-20B-0042-C100, AG-25B-0006-C100) were from Adipogen (San Diego, CA, USA). The antibody against IL-1β (Cat^#^AF-401-NA) was purchased from R&D Systems (Minneapolis, MN, USA). HRP-conjugated goat anti-rabbit IgG (H + L) (Cat^#^AS014) and α-Tubulin antibody (Cat^#^A6830) were acquired from ABclonal Biotech Co. (Wuhan, China). K^+^ turbidimetric assay kit (Cat^#^E-BC-K279-M) was from Elabscience Biotechnology Co., Ltd. (Wuhan, China). INTERFERin^®^ transfection reagent (Cat^#^101000028) was purchased from Polyplus-transfection S.A. (Illkirch, France). Mammalian protein extraction reagent (Cat^#^CW0889M) was obtained from CWBIO (Taizhou, China). MQAE Fluorescent Chloride Ion Detection Kit (Cat^#^G1725) was purchased from Wuhan Servicebio Technology Co., Ltd. (Wuhan, China). Lactate Dehydrogenase (L-LDH) Activity Assay Kit, Micromethod (Cat^#^D799208-0100) was obtained from Sangon Biotech Co., Ltd. (Shanghai, China). Mouse IL-1β ELISA kit (Cat^#^EM001-96) was acquired from ExCell Bio (Suzhou, China). FastPure Cell/Tissue DNA Isolation Mini Kit (Cat^#^DC102-01) was purchased from Vazyme Biotech Co., Ltd. (Nanjing, China). QxiSelectTM Oxidative DNA Damage ELISA Kit (Cat^#^STA-320) was obtained from Cell Biolabs, Inc. (San Diego, CA, USA). All other reagents were of analytical grade.

### 2.2. Cell Culture and Animals

The basal medium used for cell culture was DMEM containing 10% FBS and 1% penicillin/streptomycin. The J774A.1 cell line acquired from the Cell Resource Center for CAMS Institute of Basic Medical Sciences was cultured in the basal medium in a humidified incubator maintained at 37 °C and 5% CO_2_. The mouse bone marrow-derived macrophages (BMDMs) were obtained by aseptic lavage of the mouse femoral bone marrow cavity and induced to differentiate for 7 days in the above-mentioned basal medium supplemented with 40 ng/mL M-CSF.

Male ICR mice (SPF grade, 18–22 g) were acquired from Beijing Vital River Laboratory Animal Technology Co., Ltd. [Beijing, China; license number: SCXK (Beijing) 2021-0006]. All animal procedures were conducted according to the ARRIVE guidelines and were approved by the Institutional Care and Use Committee of the Institute of Medicinal Plant Development (IMPLAD) of the Chinese Academy of Medical Sciences (No. SLXD-20260228003). The experimental animals were housed in an SPF environment with controlled temperature and humidity, maintained under a 12 h light/dark cycle. During the experimental procedures, anesthesia and all other required precautions were employed to minimize animal suffering.

### 2.3. Cell Viability Assay

The PON was evaluated on J774A.1 cells (4 × 10^5^ cells per well) for cell viability using a Cell Counting Kit-8 assay. Cells were seeded into 96-well plates and treated with various concentrations of PON. After 22 h of incubation, 20 μL of CCK-8 solution was added to each well containing 200 μL of culture medium. Two hours later, a multifunctional microplate reader measured the absorbance at 450 nm.

### 2.4. Activation of Inflammasomes

To activate the NLRP3 inflammasome, J774A.1 macrophages were primed with 50 ng/mL LPS for 4 h, followed by three washes with D-Hank’s. Subsequently, cells were treated with either complete medium or PON for 30 min. Canonical NLRP3 activation was induced using the following inflammasome activators: 20 μM nigericin for 45 min, 2 mM ATP for 1 h, or 200 μg/mL MSU for 4.5 h [15].

J774A.1 cells were primed with Pam3CSK4 (400 ng/mL, 4 h) to induce non-canonical NLRP3 activation. Subsequently, cells were pretreated with PON for 30 min, followed by transfection with 1 µg/mL LPS using INTERFERin for 16 h. To assess the effects of PON on absent in melanoma-2 (AIM2) inflammasomes, the priming step used LPS (50 ng/mL, 4 h), after which cells received a 30 min PON treatment and were then transfected with poly(dA:dT) (2 µg/mL) delivered by INTERFERin for 6 h. Supernatant IL-1β was determined by the commercial ELISA kit.

### 2.5. Assessment of Pyroptosis by LDH Assay

Following stimulation, the 96-well plates were centrifuged at 350× *g* for 5 min, and the supernatants were collected for analysis. Macrophage pyroptosis was assessed by determining LDH release according to the instructions for the LDH assay kit.

### 2.6. Determination of ASC Oligomerization and ASC Speck Formation

ASC oligomerization was assessed following a previously established protocol with minor modifications [16]. Briefly, cells were washed three times with PBS and then lysed in CHAPS buffer (20 mM HEPES-KOH, 5 mM MgCl_2_, 0.5 mM EGTA, 0.1% CHAPS, supplemented with protease inhibitors). The lysates were homogenized by 20 passes through a 20-gauge needle. After centrifugation at 400× *g* for 5 min to remove nuclear debris, the resulting supernatants were subjected to further centrifugation at 6200× *g* for 10 min. The pellets containing ASC oligomers were collected, resuspended in CHAPS buffer, and incubated with 2 mM disuccinimidyl suberate for 30 min to induce cross-linking. The samples were then mixed with loading buffer and analyzed by Western blotting.

To visualize ASC speck formation, immunofluorescence staining was conducted according to a previously described procedure [17]. Briefly, 10 ng/mL LPS-primed BMDMs were pretreated with PON for 30 min and subsequently stimulated with 10 μM nigericin for 45 min. Cells were then rinsed three times with D-Hank’s solution, fixed with 4% paraformaldehyde for 15 min. After fixation, cells were incubated with quenching buffer for 2 h, washed with PBST, and blocked with blocking buffer for 1 h. Thereafter, the cells were incubated overnight at 4 °C with anti-ASC antibody (diluted 1:250), followed by Alexa Fluor 488-conjugated secondary antibody (1:250) for 1 h at room temperature. Nuclear staining was performed using Hoechst 33342. Fluorescent images were captured using a Zeiss Axio Imager.M2 fluorescence microscope (Carl Zeiss Microscopy GmbH, Jena, Germany).

### 2.7. Measurement of Intracellular K^+^

Cells were lysed on ice by sonication. The intracellular K^+^ levels in the resulting lysates were quantified using a commercial K^+^ turbidimetric assay kit in accordance with the manufacturer’s instructions.

### 2.8. Measurement of Intracellular Cl^−^

Intracellular chloride was measured using the chloride-sensitive fluorescent probe MQAE. Following the indicated treatments, the supernatant in 12-well plates was removed, and 300 μL of ddH_2_O was added to each well. After incubation at 37 °C for 15 min, the lysates were harvested and transferred to 1.5 mL EP tubes. To achieve complete cell lysis, samples were subjected to two liquid nitrogen freeze–thaw cycles, followed by centrifugation at 8000× *g* for 5 min. Thereafter, 50 μL of the clarified supernatant was mixed with an equal volume of 10 μM MQAE and transferred to a 96-well plate for fluorescence detection.

### 2.9. Measurement of Cytosolic Ca^2+^

After the indicated treatments, cells were collected and resuspended in 1 mL of Fluo-3 AM working solution, followed by incubation at 30 °C for 30 min in the dark. Probenecid was then added to a final concentration of 4 mM to prevent Fluo-3 leakage. Cells were centrifuged again, washed once with HEPES buffer, and resuspended in HEPES buffer. In a 96-well black fluorescence assay plate, the cell concentration was adjusted to 6 × 10^4^ cells per well. After addition of the indicated drugs, fluorescence was immediately monitored at Ex/Em = 485/538 nm using a fluorescence microplate reader. Nigericin was then added to a final concentration of 20 μM and fluorescence was measured immediately.

### 2.10. Measurement of mtROS

Cells were incubated with MitoSOX™ Red, a mitochondrial superoxide-specific fluorescent probe, for 30 min. Subsequently, the cells were rinsed with Hank’s buffer to remove excess probe. Fluorescence images were acquired using a Nikon ECLIPSE Ts2R-FL fluorescence microscope (Nikon Corporation, Tokyo, Japan), and fluorescence intensity was quantified using ImageJ software (version 1.53e; National Institutes of Health, Bethesda, MD, USA).

### 2.11. Measurement of ATP

Cells were lysed on ice with lysis buffer (200 μL/well in 6-well plates). After incubation for 5–10 min, the lysates were collected and centrifuged at 12,000× *g* for 5 min. A portion of the supernatant was used for protein quantification by BCA assay, and the remainder was used for ATP measurement according to the manufacturer’s instructions. Luminescence was recorded using a Fluoroskan Ascent FL chemiluminescence analyzer (Thermo Fisher Scientific, Madison, WI, USA), and ATP levels were normalized to total protein concentration.

### 2.12. Measurement of Mitochondrial Membrane Potential

To evaluate mitochondrial membrane potential, cells were stained with JC-1. Briefly, cells were processed with an enhanced mitochondrial membrane potential detection kit in accordance with the manufacturer’s protocol, with carbonyl cyanide m-chlorophenylhydrazone (CCCP) serving as the positive control. Fluorescence intensity was measured with a Synergy H1 fluorescence microplate reader (BioTek Instruments, Inc., Winooski, VT, USA), and the membrane potential was quantified by calculating the red-to-green fluorescence intensity ratio.

### 2.13. Isolation and Quantification of Cytosolic mtDNA and ox-mtDNA

Cells were equally divided into two 1.5 mL microcentrifuge tubes for the isolation of total cellular DNA and cytosolic DNA, respectively. After centrifugation at 200× *g* for 5 min, the pellet designated for cytosolic DNA extraction was gently resuspended in Buffer A (150 mM NaCl, 50 mM HEPES, and 25 μg/mL digitonin, pH 7.4) and incubated at room temperature for 10 min to selectively permeabilize the plasma membrane. The samples were then centrifuged at 1000× *g* for 10 min, and the supernatants were transferred to fresh tubes and further centrifuged at 17,000× *g* for 10 min to remove residual cellular debris and organelles. The resulting supernatants were collected as the cytosolic fraction [18].

DNA from the whole-cell fraction and cytosolic fraction was extracted using a FastPure Cell/Tissue DNA Isolation Mini Kit according to the manufacturer’s instructions. Cytosolic mtDNA was quantified by qPCR using primers specific for the mitochondrial D-loop region, with TERT used as the nuclear reference (Table 1). The relative cytosolic mtDNA level was expressed as the normalized D-loop/TERT ratio. Cytosolic ox-mtDNA was assessed by measuring 8-OH-dG levels in cytosolic DNA using an OxiSelect Oxidative DNA Damage ELISA Kit according to the manufacturer’s instructions.

### 2.14. Western Blot Analysis

GSDMD, NLRP3, and ASC proteins were extracted from whole-cell lysates using a commercial mammalian protein extraction kit. Supernatant proteins, including caspase-1 p20 and IL-1β p17, were prepared with minor modifications based on a previously described method [19]. Briefly, culture supernatants were collected and centrifuged at 350× *g* for 5 min to remove residual cells and cellular debris. Proteins in the clarified supernatants were then precipitated by methanol/chloroform extraction. After phase separation, the protein interphase was washed twice with cold methanol. The resulting protein pellets were briefly air-dried, resuspended in 1× SDS loading buffer, and boiled for 5 min. The final supernatants were collected for subsequent SDS-PAGE.

Protein concentrations in whole-cell lysates were quantified using a BCA assay kit. Equal quantities of protein were resolved on SDS–PAGE gels and electrotransferred to PVDF membranes. Following blocking with 5% nonfat milk for 2 h at room temperature, the membranes were incubated overnight at 4 °C with the appropriate primary antibodies. After three washes with TBST, the membranes were incubated with HRP-conjugated secondary antibodies for 2 h. Immunoreactive bands were visualized using an enhanced chemiluminescence detection kit and captured with a ChemiScope Capture system (CLINX, Shanghai, China). Band intensities were quantified using ImageJ software (version 1.52a; National Institutes of Health, Bethesda, MD, USA), and the expression levels of target proteins were normalized to the corresponding housekeeping proteins.

### 2.15. Endotoxemia Mouse Model

PON was prepared in normal saline supplemented with 5% DMSO and 2% Tween-80. For IL-1β determination, male ICR mice were randomly allocated into a negative control group, an LPS-treated model group (5 mg/kg), and PON-treated groups (10 and 20 mg/kg). PON was administered intraperitoneally 30 min before intravenous LPS challenge, whereas mice in the negative control and model groups received the same volume of vehicle. Three hours after LPS challenge, mice were anesthetized, and blood as well as peritoneal lavage fluid were collected. IL-1β levels were determined using ELISA kits in accordance with the manufacturer’s instructions.

For the determination of other proinflammatory cytokines, male ICR mice were randomly assigned to a negative control group, an LPS model group (1 mg/kg), and PON-treated groups (5 and 10 mg/kg). The drug administration and modeling procedures were the same as those described above. Blood samples were collected 2 h after LPS administration, and serum TNF-α and MCP-1 levels were quantified by ELISA.

### 2.16. MSU-Induced Peritonitis Mouse Model

MSU crystals were prepared as previously described [20]. Mice were randomly assigned to four groups: normal control group, model group (2 mg/mouse MSU crystals), and PON treatment groups (10 or 20 mg/kg). PON was administered intraperitoneally before MSU stimulation, whereas mice in the normal control and model groups received an equal volume of vehicle. After 30 min, peritonitis was induced by intraperitoneal injection of MSU crystals (2 mg/mouse). Six hours after the MSU challenge, the mice were euthanized, and the peritoneal cavity was lavaged with 1.5 mL of ice-cold PBS. The level of IL-1β in the peritoneal lavage fluid was measured using a commercial ELISA kit.

### 2.17. Statistical Analysis

All experiments were repeated at least three times and results were expressed as mean ± SD from a representative experiment. Statistical analyses were carried out using GraphPad Prism (version 9.5.1). A Student’s *t*-test was used for comparing two groups, and one-way ANOVA followed by the Tukey posttest was used for comparing more than two groups. *p* < 0.05 was considered statistically significant.

## 3. Results

### 3.1. PON Suppresses NLRP3 Agonist-Induced Maturation of IL-1β and Caspase-1 in LPS-Primed J774A.1 Cells

**Figure 1:**
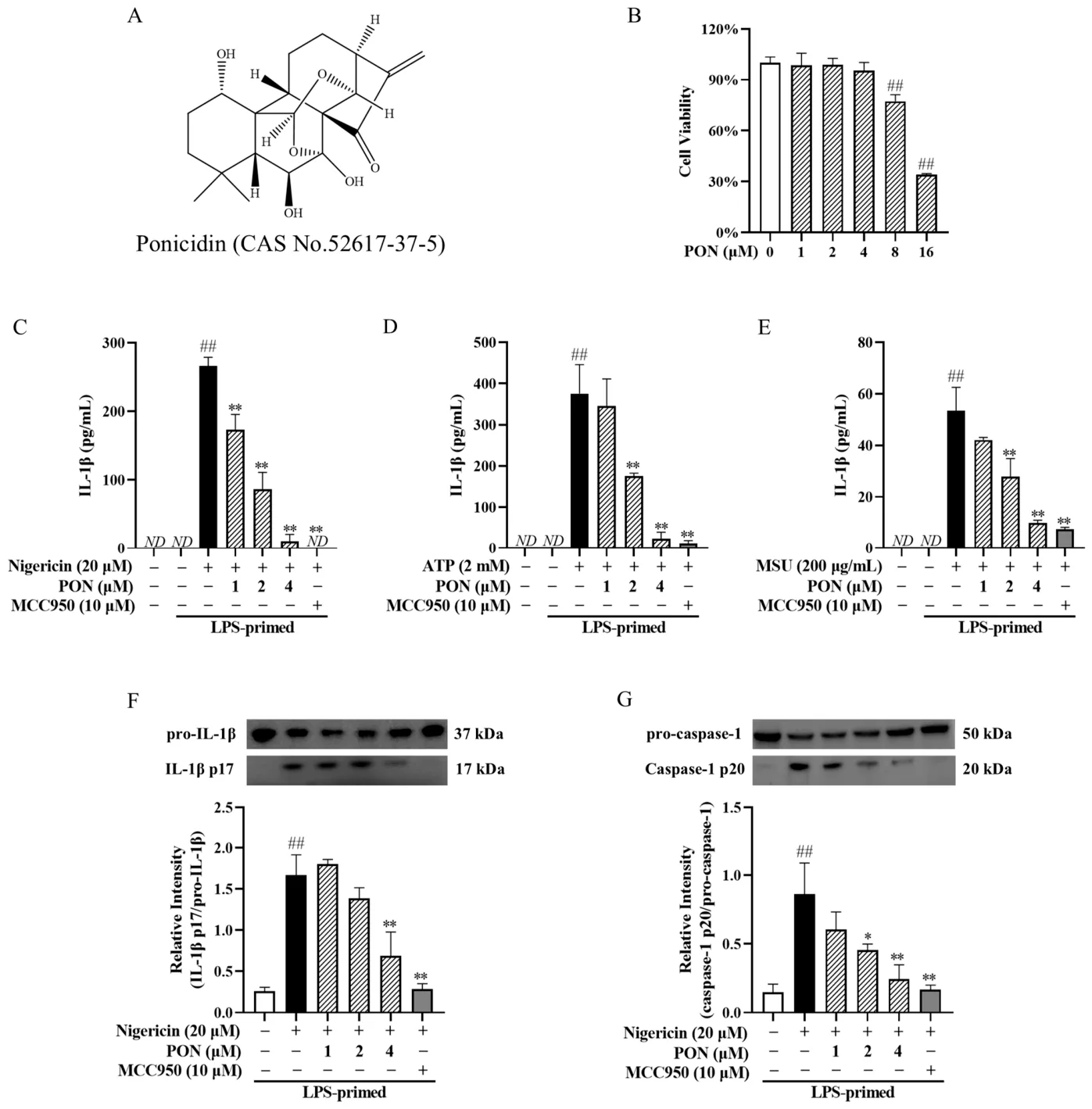
PON inhibits IL-1β and caspase-1 cleavage induced by NLRP3 agonists in J774A.1 cells. (**A**) PON structure. (**B**) Effect of PON on the viability of J774A.1 cells. (**C**–**E**) Effects of PON on supernatant IL-1β in J774A.1 cells. The cells were primed with LPS for 4 h and then treated with PON for 30 min, prior to stimulation with nigericin for 45 min, ATP for 1 h, or MSU crystals for 4.5 h. MCC950 was used as a positive control. ^##^ *p* < 0.01 vs. LPS alone; ** *p* < 0.01 vs. LPS + nigericin (**C**) or ATP (**D**) or MSU (**E**). ND, not detected. (**F**) Effect of PON on pro-IL-1β levels in whole cell lysates and mature IL-1β p17 levels in the supernatant. ^##^ *p* < 0.01 vs. LPS alone; ** *p* < 0.01 vs. LPS + nigericin. (**G**) Effect of PON on pro-caspase-1 levels in whole cell lysates and activated caspase-1 p20 levels in the supernatant. ^##^ *p* < 0.01 vs. LPS alone; * *p* < 0.05 and ** *p* < 0.01 vs. LPS + nigericin.

To ensure that the inhibitory effects of PON were not attributable to cytotoxicity, we first assessed its impact on viability in J774A.1 macrophages. The CCK8 assay demonstrated that PON had no significant effect on cell viability at concentrations up to 4 μM. Thus, the non-toxic concentrations (1–4 µM) were selected for subsequent assessment (Figure 1A,B). To evaluate whether PON could block NLRP3 activation, we treated LPS-primed J774A.1 cells with PON for 30 min before stimulation and measured IL-1β secretion. As shown in Figure 1C–E, PON concentration-dependently suppressed supernatant IL-1β levels induced by three NLRP3 agonists (nigericin, ATP, or MSU), suggesting PON as a broad-spectrum inhibitor of the NLRP3 signal.

Supernatant mature IL-1β is generated from the cleavage of pro-IL-1β by activated caspase-1. Therefore, the ratio of mature IL-1β in the supernatant to intracellular pro-IL-1β reflects the extent of caspase-1 activity. Indeed, PON concentration-dependently reduced the mature IL-1β (p17)/pro-IL-1β (p37) ratio (Figure 1F), suggesting that PON interferes with caspase-1-mediated cleavage of pro-IL-1β. To verify this possibility, we measured the ratio of active caspase-1 in the supernatant to inactive pro-caspase-1 in cell lysates. Consistently, PON concentration-dependently reduced the active caspase-1 (p20)/pro-caspase-1 (p50) ratio (Figure 1G), indicating that PON suppresses supernatant IL-1β levels by inhibiting caspase-1 activation.

### 3.2. PON Inhibits Pyroptosis Triggered by Nigericin and ATP in LPS-Primed J774A.1 Cells

**Figure 2:**
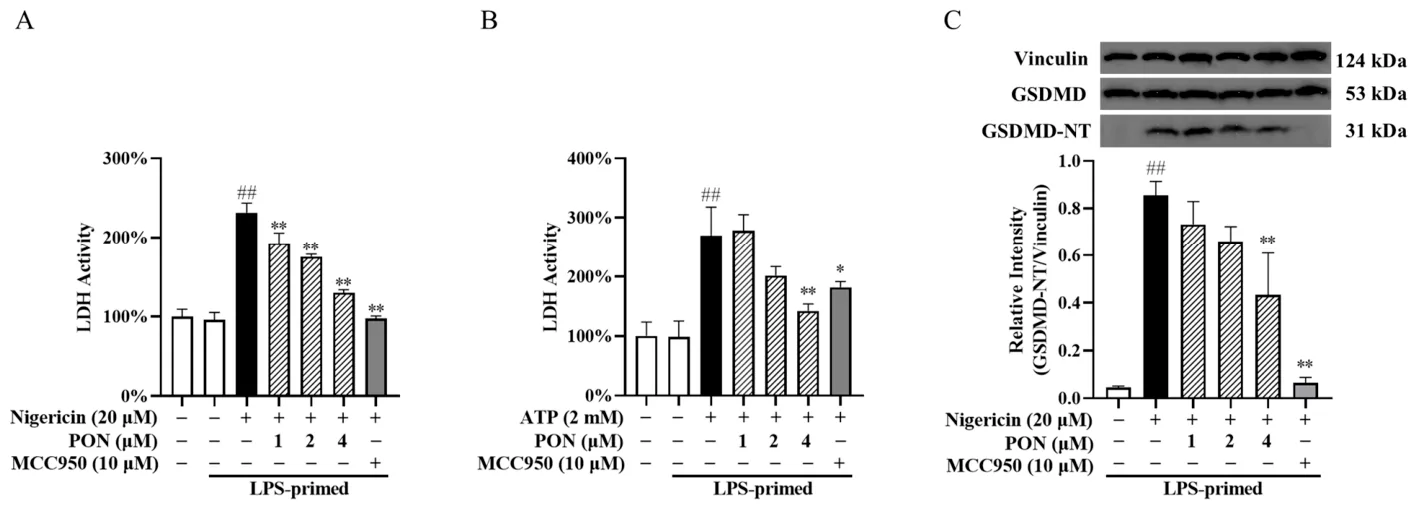
PON selectively inhibits canonical NLRP3-dependent pyroptosis. (**A**) Effect of PON on supernatant LDH induced by nigericin in J774A.1 cells. ^##^ *p* < 0.01 vs. LPS alone; ** *p* < 0.01 vs. LPS + nigericin. (**B**) Effect of PON on supernatant LDH induced by ATP in J774A.1 cells. ^##^ *p* < 0.01 vs. LPS alone; * *p* < 0.05 and ** *p* < 0.01 vs. LPS + ATP. (**C**) Effect of PON on GSDMD cleavage. ^##^ *p* < 0.01 vs. LPS alone; ** *p* < 0.01 vs. LPS + nigericin.

Unlike the unique mode of macrophage cell death induced by MSU crystals [21,22], nigericin- and ATP-triggered cell death is mediated by GSDMD-dependent pyroptosis, a hallmark downstream event of NLRP3 inflammasome activation. Cleaved GSDMD forms pores in the plasma membrane, leading to loss of membrane integrity and LDH release, and thus LDH levels in the supernatant reflect the extent of pyroptosis. To further confirm the inhibitory effect of PON on NLRP3 inflammasome activation, its effects on pyroptosis were evaluated using an LDH release assay. As shown in Figure 2A,B, stimulation of LPS-primed J774A.1 macrophages with nigericin or ATP led to a marked increase in LDH release, while PON treatment significantly reduced LDH levels in the supernatant. Consistently, PON suppressed nigericin-induced GSDMD cleavage (Figure 2C), indicating that PON attenuated nigericin- or ATP-triggered pyroptosis in LPS-primed J774A.1 cells.

### 3.3. PON Inhibits Non-Canonical NLRP3 Activation Without Affecting AIM2 Inflammasome

**Figure 3:**
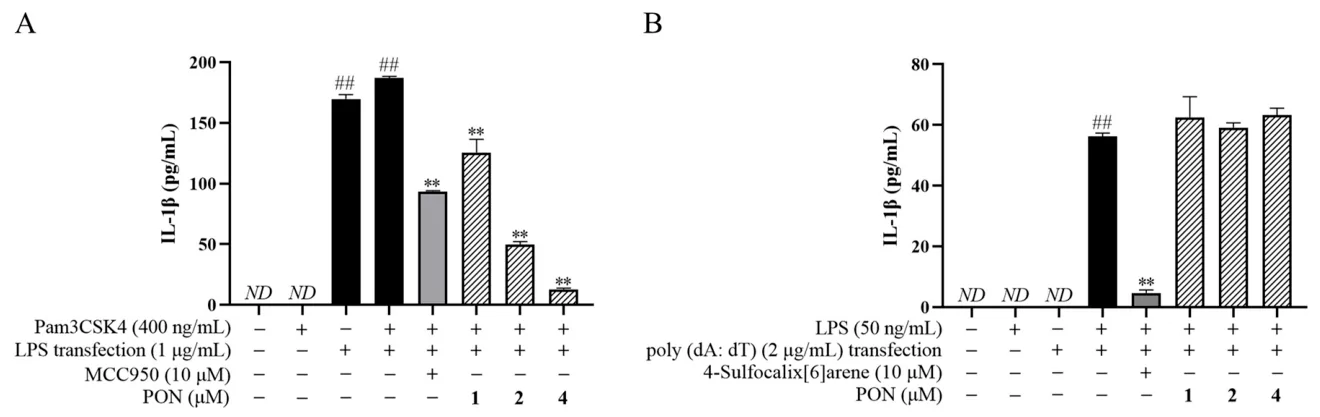
Effects of PON on non-canonical NLRP3 and AIM2 inflammasome activation. (**A**) Effect of PON on IL-1β levels in cell supernatants upon activation of the non-canonical inflammasome. ^##^ *p* < 0.01 vs. Pam3CSK4 alone. ** *p* < 0.01 vs. Pam3CSK4 + LPS transfection. (**B**) Effect of PON on IL-1β levels in cell supernatants upon activation of the AIM2 inflammasome. ^##^ *p* < 0.01 vs. LPS alone; ** *p* < 0.01 vs. LPS + poly (dA:dT) transfection. ND, not detected.

Besides the canonical route, the processing of NLRP3-dependent IL-1β may also be activated indirectly through a non-canonical pathway that operates downstream of caspase-11 [23]. Thus, we evaluated the effect of PON on the non-canonical pathway. J774A.1 cells were first primed with Pam3CSK4 (a TLR1/2 ligand) and then pretreated with PON before stimulation with cytosolic LPS (cLPS). The results showed that stimulation of Pam3CSK4-primed J774A.1 macrophages with cLPS led to a robust increase in supernatant IL-1β, while PON concentration-dependently reduced IL-1β levels, indicating that PON can also dampen the non-canonical pathway (Figure 3A).

To determine whether the inhibitory effects of PON were specific to the NLRP3 inflammasome, we next investigated its effect on the AIM2 inflammasome complex, which shares the same downstream signaling components (ASC and caspase-1) despite utilizing a distinct sensor protein. LPS-primed J774A.1 cells were pretreated with PON and then stimulated with poly (dA:dT), an AIM2 activator. Poly (dA:dT) induced a marked IL-1β release in LPS-primed J774A.1 macrophages, but PON pretreatment did not affect this release (Figure 3B). These findings demonstrate that PON suppresses NLRP3 rather than AIM2 inflammasome activation, even though the activation of both AIM2 and canonical NLRP3 inflammasomes recruits ASC as a common adaptor for caspase-1 activation.

### 3.4. PON Inhibits ASC Oligomerization and Speck Formation

**Figure 4:**
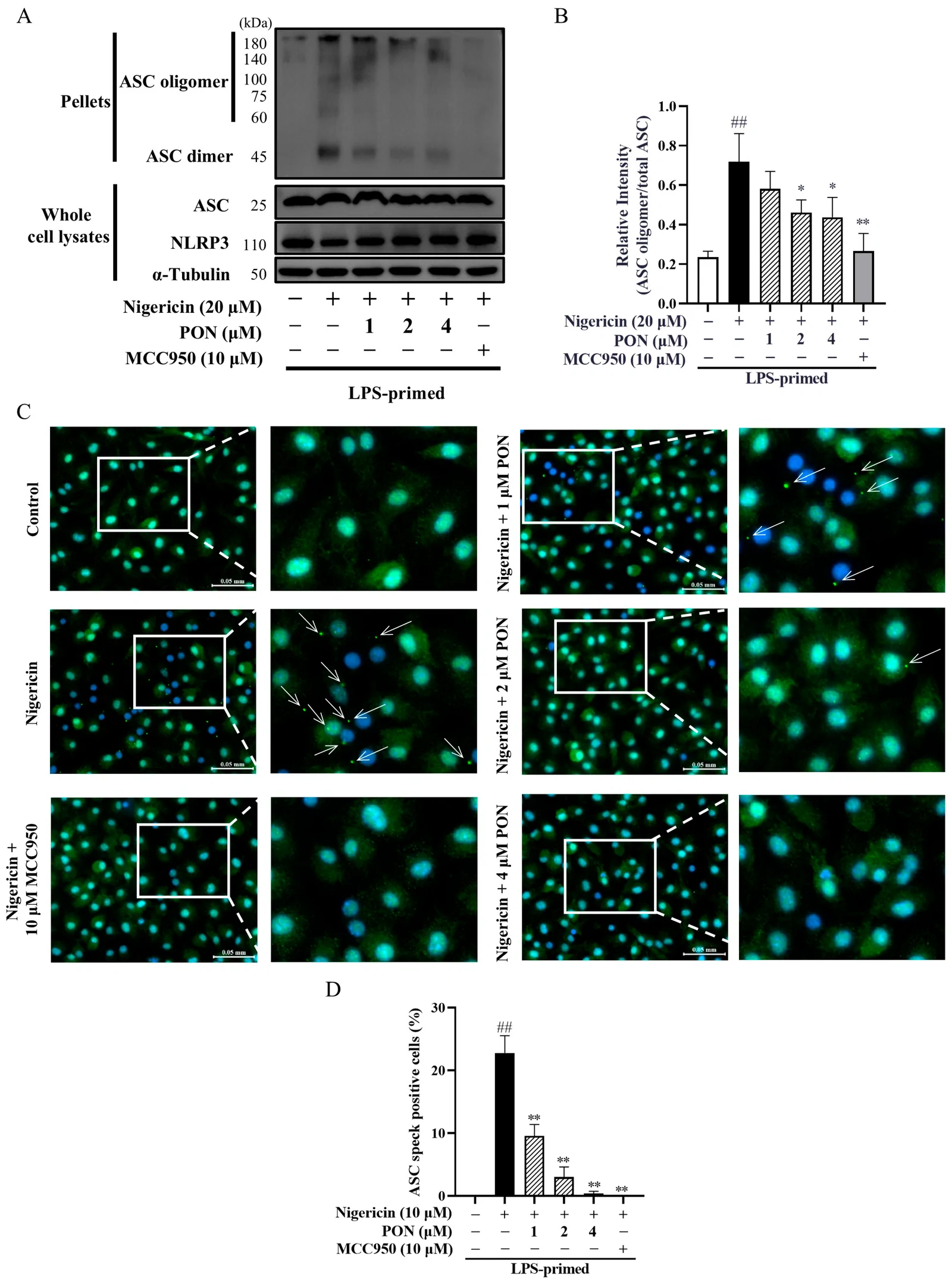
Effects of PON on ASC oligomerization and speck formation. (**A**) Representative Western blot images of J774A.1 cells showing the effects of PON on ASC oligomers in the pellets, as well as on ASC, NLRP3 and α-tubulin in the whole cell lysates. (**B**) Effect of PON on ASC oligomer intensity in the pellets. (**C**) Representative immunofluorescence images of BMDMs showing the subcellular distribution of ASC (green), with specks appearing as bright punctate structures. Hoechst 33342 (blue) was used to stain nuclei. White arrows indicate the ASC specks. Scale bar, 0.05 mm. (**D**) Effect of PON on the percentage of cells with ASC specks. At least three fields and more than 100 cells were counted for each condition. ^##^ *p* < 0.01 vs. LPS alone; * *p* < 0.05 and ** *p* < 0.01 vs. LPS + nigericin.

During canonical inflammasome activation, NLRP3 interacts with ASC to promote the oligomerization of ASC, which forms distinct speck-like structures essential for recruiting and activating pro-caspase-1. Considering that PON did not affect the expression of NLRP3 and ASC in LPS-primed J774A.1 cells (Figure 4A), it is reasonable to hypothesize that PON functions by disrupting the assembly of the NLRP3 inflammasome. We therefore evaluated whether PON influences nigericin-triggered ASC oligomerization and speck formation. As illustrated in Figure 4A,B, PON significantly suppressed ASC oligomerization induced by nigericin. To visually confirm this disruption in assembly, ASC speck formation was assessed by immunofluorescence in BMDMs. In LPS-primed BMDMs, ASC exhibited a diffuse distribution under resting conditions but formed prominent green puncta upon nigericin challenge, while PON substantially reduced the formation of such specks (Figure 4C,D). These results indicate that PON inhibits nigericin-induced NLRP3 inflammasome assembly, as evidenced by reduced ASC oligomerization and speck formation.

### 3.5. PON Has No Effect on K^+^, Cl^−^ or Ca^2+^ Flux During NLRP3 Inflammasome Activation

**Figure 5:**
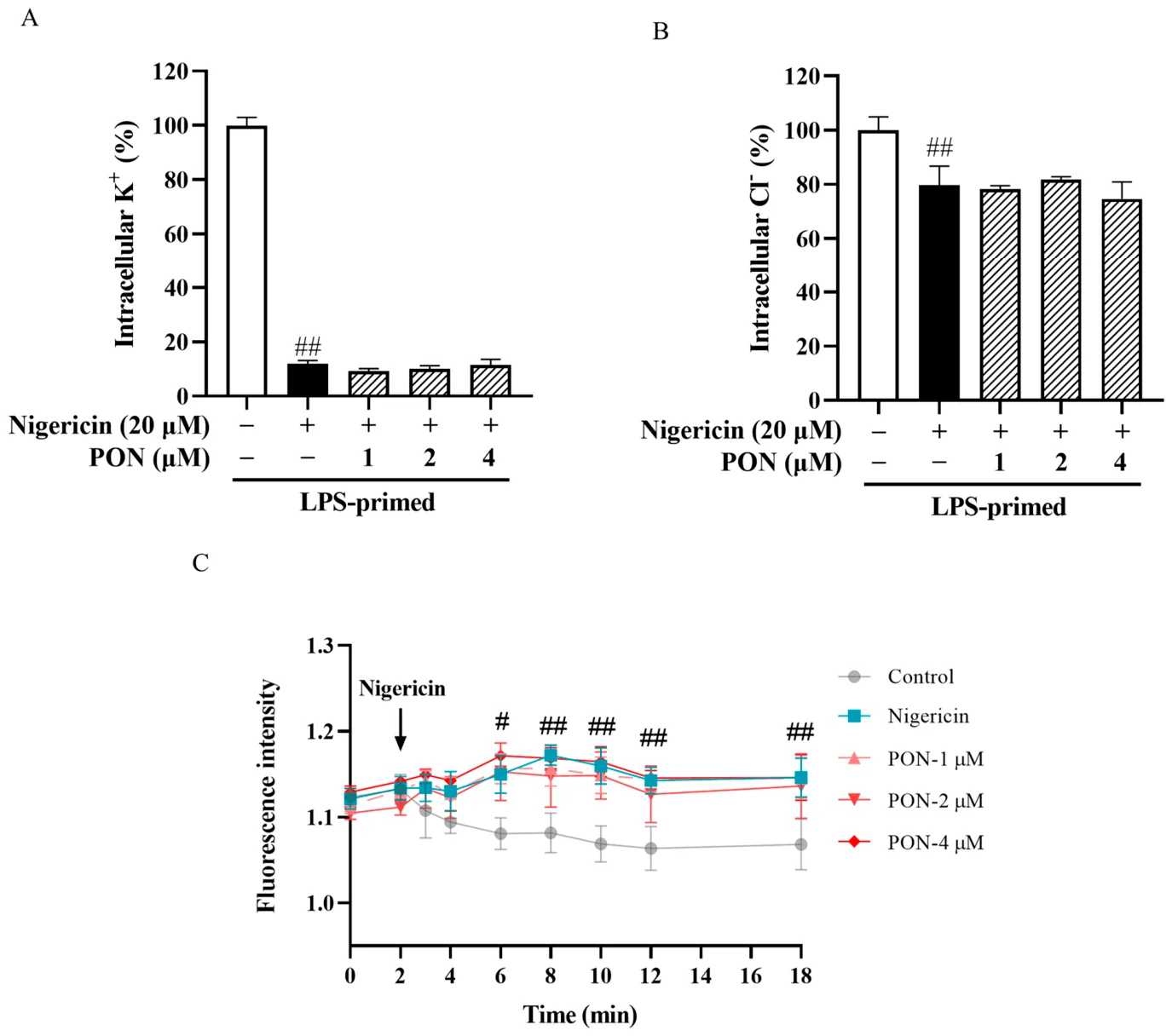
Effects of PON on cellular ion flux in J774A.1 cells. (**A**) Effect of PON on K^+^ efflux. (**B**) Effect of PON on Cl^−^ efflux. (**C**) Effect of PON on cytosolic Ca^2+^ mobilization. ^##^ *p* < 0.01 vs. LPS alone. ^#^: *p* < 0.05.

Next, we investigated the effects of PON on K^+^ and Cl^−^ efflux, as well as Ca^2+^ influx, which contribute to NLRP3 inflammasome assembly and activation [24]. Nigericin significantly decreased the intracellular concentrations of K^+^ and Cl^−^ in LPS-primed J774A.1 cells (Figure 5A,B), whereas PON had no impact on the efflux of these ions. Regarding Ca^2+^ flux, nigericin induced a significant increase in cytosolic Ca^2+^, while PON also had no impact on this event (Figure 5C). These data demonstrate that PON inhibits NLRP3 inflammasome activation independent of any alterations in K^+^, Cl^−^, or Ca^2+^ flux.

### 3.6. PON Attenuates Mitochondrial Damage During NLRP3 Inflammasome Activation

**Figure 6:**
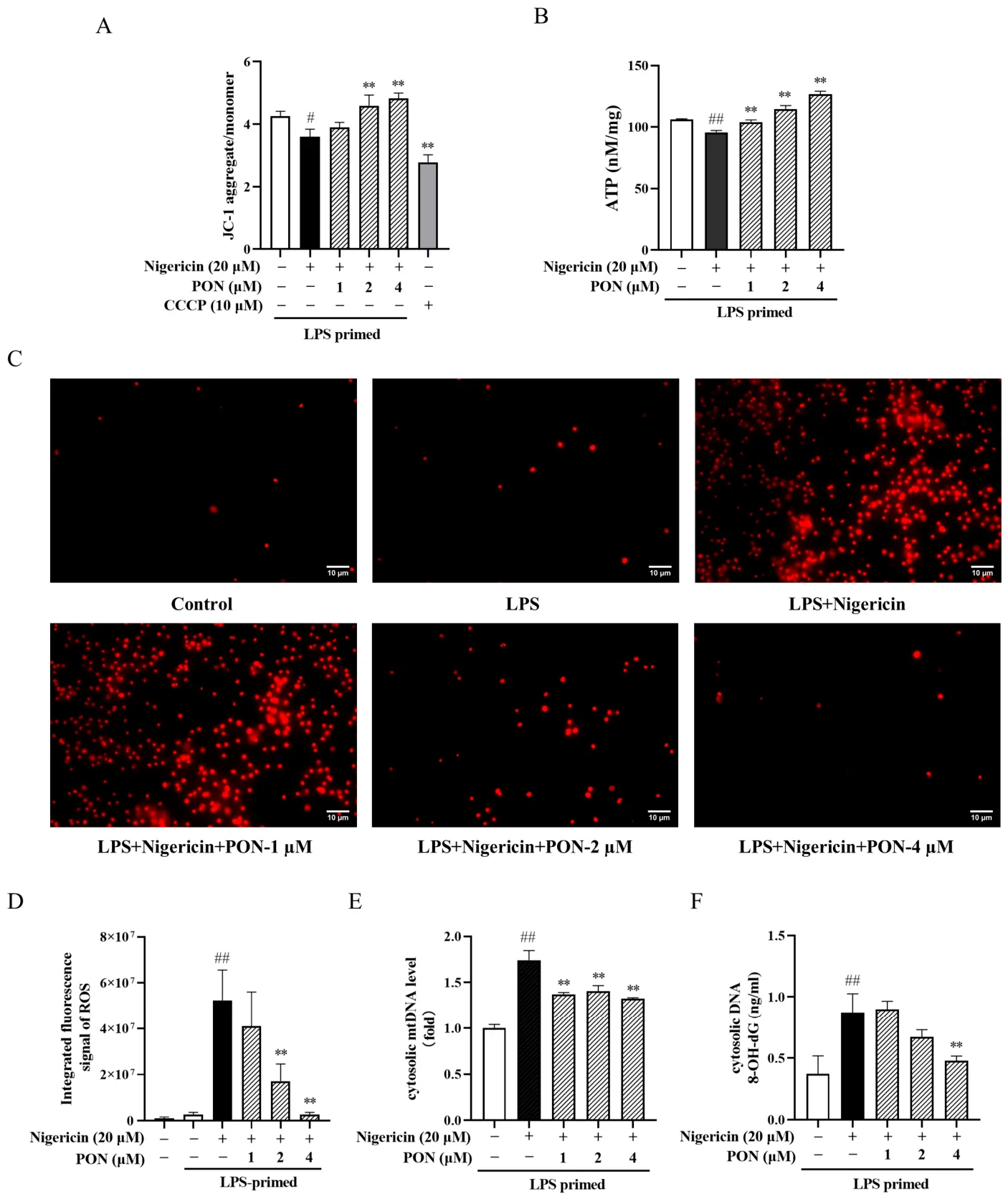
Effects of PON on mitochondrial dysfunction (*n* = 3). (**A**) Effect of PON on membrane potential in J774A.1 cells. CCCP was used as a positive control to dissipate the mitochondrial membrane potential. (**B**) Effect of PON on ATP levels in J774A.1 cells. (**C**) Representative immunofluorescence images of mtROS in J774A.1 cells. Scale bar, 10 μm. (**D**) Effect of PON on integrated fluorescence intensity of mtROS. At least three fields and more than 100 cells were counted for each condition. (**E**) Effect of PON on cytosolic mtDNA levels in J774A.1 cells. (**F**) Effect of PON on cytosolic ox-mtDNA levels in J774A.1 cells. ^#^ *p* < 0.05 vs. LPS alone; ^##^ *p* < 0.01 vs. LPS alone; ** *p* < 0.01 vs. LPS + nigericin.

Given that PON had no effect on ion fluxes, we next turned our attention to mitochondria, a critical platform for NLRP3 inflammasome assembly and activation. As the hubs of the respiratory chain and oxidative phosphorylation complexes, damaged mitochondria are characterized by a loss of membrane potential and decreased ATP production. Mitochondrial dysfunction is further accompanied by the release of mitochondrial DAMPs, which contribute to NLRP3 inflammasome activation. Therefore, we next examined whether PON affected nigericin-induced mitochondrial dysfunction. As shown in Figure 6A,B, nigericin stimulation caused a significant decrease in mitochondrial membrane potential and intracellular ATP levels, whereas PON effectively prevented membrane potential loss and restored ATP levels.

We further investigated whether PON affected mtROS accumulation, mtDNA release, and cytosolic oxidized mtDNA levels. As shown in Figure 6C,D, Nigericin markedly increased mtROS production, while PON concentration-dependently reduced mtROS levels. Consistently, nigericin also induced mtDNA leakage into the cytoplasm, whereas PON significantly decreased this leakage (Figure 6E). Moreover, PON also mitigated the oxidative modification of mtDNA, as determined by 8-OH-dG detection (Figure 6F). These results indicate that PON alleviates nigericin-induced mitochondrial dysfunction.

### 3.7. PON Exerts Anti-Inflammatory Effects In Vivo

**Figure 7:**
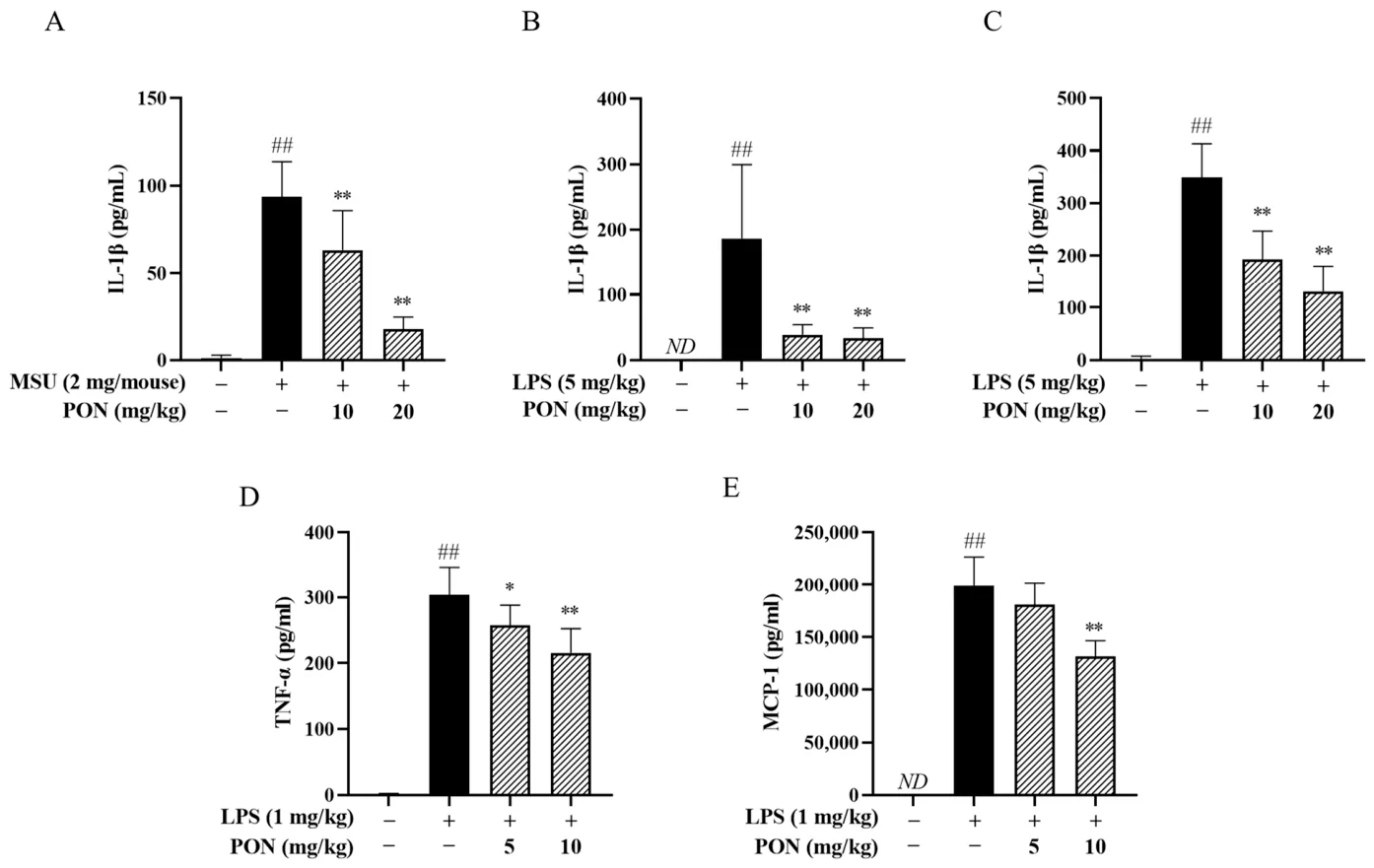
Effects of PON on MSU- and LPS-induced inflammatory responses in vivo. (**A**) Effect of PON on IL-1β levels in peritoneal lavage fluid of MSU-induced peritonitis mice (*n* = 9). ^##^ *p* < 0.01 vs. negative control; ** *p* < 0.01 vs. MSU alone. (**B**,**C**) Effects of PON on IL-1β levels in the peritoneal lavage fluid (**B**) and serum (**C**) of endotoxemia mice (*n* = 8). (**D**,**E**) Effects of PON on serum TNF-α (**D**) and MCP-1 (**E**) levels in endotoxemia mice (*n* = 8). ^##^  *p* < 0.01 vs. negative control; * *p* < 0.05 and ** *p* < 0.01 vs. LPS alone. ND, not detected.

To further determine whether PON suppresses NLRP3 inflammasome activation in vivo, two mouse models of inflammation were employed. MSU-induced peritonitis is a classical in vivo model of NLRP3 inflammasome activation, where MSU crystals stimulate peritoneal macrophages to activate the inflammasome and enhance IL-1β secretion. As shown in Figure 7A, MSU stimulation markedly increased IL-1β levels in peritoneal lavage fluid, while PON significantly reduced IL-1β production, with inhibitory rates of 32.5% and 81.6%.

In endotoxemia, LPS induces systemic inflammation through TLR4-dependent priming of monocytes/macrophages and also contributes to NLRP3 inflammasome activation in vivo [25]. The effects of PON on proinflammatory mediators were next investigated. As shown in Figure 7B,C, intravenous injection of LPS robustly elevated IL-1β levels in both the peritoneal lavage fluid and serum, while PON markedly suppressed this elevation. In addition, serum TNF-α and MCP-1 levels were also robustly elevated in response to LPS stimulation, while PON significantly reduced their levels (Figure 7D,E). These results demonstrate that PON exerts anti-inflammatory effects in endotoxemia, which are not limited to the suppression of NLRP3 inflammasome activation.

## 4. Discussion

NLRP3 is a cytosolic receptor that senses sterile and infectious insults, thus having implications across the disease spectrum by driving innate immune responses [26]. Its activation requires two steps: the priming signal upregulates the expression of necessary elements such as NLRP3 and pro-IL-1β, and the activation signal promotes NLRP3 oligomerization and inflammasome assembly. A previous study found that PON could decrease non-NLRP3 inflammasome-dependent pro-inflammatory cytokines’ production (e.g., TNF-α, IL-6) in LPS-primed macrophages [13]. Our results also indicated that it effectively reduced serum TNF-α and MCP-1 levels in endotoxemic mice (Figure 7B–E) and decreased pro-IL-1β levels in LPS-stimulated RAW264.7 cells by inhibiting NF-κB signaling (Figures S1 and S2), further supporting its suppressive effect on the priming stage. Regarding the activation stage, PON not only reduced IL-1β release induced by multiple NLRP3 activators, but also weakened caspase-1 activation and downstream pyroptosis in LPS-primed J774A.1 cells (Figure 1 and Figure 2). In MSU-induced peritonitis mice, a single treatment of PON (10–20 mg/kg, i.p.) effectively lowered IL-1β levels in the peritoneal lavage fluid (Figure 7A). These results demonstrate that PON significantly inhibits both the priming and activation signals of NLRP3 in vivo and in vitro.

Upon sensing intracellular DAMPs, NLRP3 undergoes self-oligomerization, thus leading to pyrin domain (PYD) clustering and recruitment of the adaptor molecule ASC, which in turn interacts with the effector protein pro-caspase-1 and forms a complex (the NLRP3 inflammasome) at the perinuclear region. During this process, ASC self-assembles into helical filaments that converge to form a prominent supramolecular focus termed the ASC speck, a hallmark of NLRP3 inflammasome assembly and activation. By virtue of its fluorescent property [27], the ASC speck has become a representative indicator for NLRP3 inflammasome assembly and activation. PON effectively suppressed nigericin-triggered ASC oligomerization and speck formation (Figure 4), indicating its capacity to block NLRP3 inflammasome assembly and subsequent activation.

Given the diverse structural nature of exogenous NLRP3 agonists, it is more likely that NLRP3 senses these activators via some intermediate factors, rather than through direct interaction [28]. The movement of K^+^, Cl^−^, and Ca^2+^ across the cell membrane is the critical intermediate event, which triggers the assembly and activation of the inflammasome complex [29]. Nevertheless, our results showed that PON did not affect any of these three ion fluxes (Figure 5). Therefore, the next study placed emphasis on mitochondria which can influence NLRP3 assembly through multiple mechanisms. mtROS, the by-product of the damaged mitochondrial respiratory chain, modulates NLRP3 inflammasome activation indirectly through a ROS-sensing molecule that binds to NLRP3. A recent study found that cofilin-1, an actin-severing protein, regulates NLRP3 inflammasome by sensing mtROS. Under resting conditions, cofilin-1 directly binds to NLRP3 to prevent its activation; upon mtROS oxidation, the sequestered NLRP3 can dissociate from cofilin-1, thereby initiating assembly [30]. Moreover, mtDNA is indispensable for NLRP3 recruitment to the outer mitochondrial membrane to initiate assembly [31]; meanwhile, mtDNA is highly susceptible to oxidation into ox-mtDNA, which upon release into the cytoplasm directly binds to NLRP3 and promotes inflammasome activation [32]. Consistently, PON prominently suppressed mtROS generation and reduced cytosolic mtDNA/ox-mtDNA, accompanied by restored ATP levels [8] and membrane potential, collectively indicating an improvement in global mitochondrial function (Figure 6). Thus, PON inhibits NLRP3 assembly by protecting against global mitochondrial dysfunction, rather than by targeting a single mitochondrial event.

Taken together, these in vivo (MSU-induced peritonitis model) and in vitro results demonstrate that PON effectively inhibits NLRP3 inflammasome signaling. During the activation stage, PON prevents the decline of mitochondrial membrane potential and ATP depletion, and suppresses the excessive generation of DAMPs such as mtROS and mtDNA/ox-mtDNA. By virtue of these protective effects on mitochondria, PON suppresses downstream ASC polymerization, thereby restricting the formation of the proteolytically active inflammasome complex and reducing the cleavage of pro-IL-1β and GSDMD. Our findings may provide a promising candidate for the treatment of NLRP3 inflammasomopathies.

## 5. Limitations and Future Perspectives

Although the present study has demonstrated the protective effect of PON on mitochondrial function, the detailed underlying mechanisms remain unclear. In fact, maintaining mitochondrial homeostasis is a multi-level regulatory network centered on the mitochondrial quality control (MQC) system, including protein quality control, biogenesis, dynamics (fusion and fission), and mitophagy [33,34]. The operation of this system is precisely regulated by intracellular and extracellular signals, such as AMPK, sirtuins, metabolites, as well as redox signals. Which specific steps involved in the protective effects of PON on mitochondrial health remain to be further elucidated.

## Figures and Tables

**Table 1:** Primer sequences used in qPCR analysis of cytosolic mtDNA.

Gene		Primer Sequences
D-loop	F	5′-AAT CTA CCA TCC TCC GTG AAA CC-3′
	R	5′-TCA GTT TAG CTA CCC CCA AGT TTA A-3′
TERT	F	5′-CTA GCT CAT GTG TCA AGA CCC TCT T-3′
	R	5′-GCC AGC ACG TTT CTC TCG TT-3′

F: forward primer; R: reverse primer.

## Data Availability

The original contributions presented in this study are included in the article/Supplementary Material. Further inquiries can be directed to the corresponding authors.

## Supplementary Materials

The following supporting information can be downloaded at: https://www.mdpi.com/article/10.3390/antiox15101325/s1, Table S1. Primer sequences used in RT-qPCR analysis of IL-1β; Figure S1: PON suppresses pro-IL-1β in LPS-stimulated RAW264.7 cells; Figure S2: PON inhibits NF-κB pathway in LPS-stimulated RAW264.7 cells; File S1. RAW Datas.

## Abbreviations

The following abbreviations are used in this manuscript:

AIM2	absent in melanoma-2
ASC	apoptosis-associated speck-like protein containing a caspase recruitment domain
BMDMs	bone marrow-derived macrophages
CCCP	carbonyl cyanide m-chlorophenylhydrazone
cLPS	cytosolic lipopolysaccharide
DAMPs	damage-associated molecular patterns
GSDMD	gasdermin D
LDH	lactate dehydrogenase
MSU	monosodium urate
mtDNA	mitochondrial DNA
mtROS	mitochondrial reactive oxygen species
NLRP3	NOD-like receptor family pyrin domain-containing 3
ox-mtDNA	oxidized mitochondrial DNA
PAMPs	pathogen-associated molecular patterns
PON	ponicidin
PRR	pattern recognition receptor
PYD	pyrin domain
*R. rubescens*	*Rabdosia rubescens* (Hemsl.) Hara

## References

[1] Ortega M.A., De Leon-Oliva D., García-Montero C., Fraile-Martinez O., Boaru D.L., de Castro A.V., Saez M.A., Lopez-Gonzalez L., Bujan J., Alvarez-Mon M.A. (2023). Reframing the link between metabolism and NLRP3 inflammasome: Therapeutic opportunities. Front. Immunol..

[2] Barnett K.C., Li S., Liang K., Ting J.P. (2023). A 360° view of the inflammasome: Mechanisms of activation, cell death, and diseases. Cell.

[3] Seoane P.I., Lee B., Hoyle C., Yu S., Lopez-Castejon G., Lowe M., Brough D. (2020). The NLRP3-inflammasome as a sensor of organelle dysfunction. J. Cell. Biol..

[4] Li N., Zhang R., Tang M., Zhao M., Jiang X., Cai X., Ye N., Su K., Peng J., Zhang X. (2023). Recent Progress and Prospects of Small Molecules for NLRP3 Inflammasome Inhibition. J. Med. Chem..

[5] Liu Q., Zhang D., Hu D., Zhou X., Zhou Y. (2018). The role of mitochondria in NLRP3 inflammasome activation. Mol. Immunol..

[6] Zhang T., Zhao J., Liu T., Cheng W., Wang Y., Ding S., Wang R. (2022). A novel mechanism for NLRP3 inflammasome activation. Metabol. Open..

[7] Bronner D.N., Abuaita B.H., Chen X., Fitzgerald K.A., Nuñez G., He Y., Yin X.M., O’Riordan M.X. (2015). Endoplasmic Reticulum Stress Activates the Inflammasome via NLRP3- and Caspase-2-Driven Mitochondrial Damage. Immunity.

[8] Yang S., Huang G., Ting J.P. (2025). Mitochondria and NLRP3: To die or inflame. Immunity.

[9] Sarwar M.S., Xia Y.X., Liang Z.M., Tsang S.W., Zhang H.J. (2020). Mechanistic Pathways and Molecular Targets of Plant-Derived Anticancer ent-Kaurane Diterpenes. Biomolecules.

[10] Ali M.A., Khan N., Ali A., Akram H., Zafar N., Imran K., Khan T., Khan K., Armaghan M., Palma-Morales M. (2024). Oridonin from Rabdosia rubescens: An emerging potential in cancer therapy—A comprehensive review. Food. Sci. Nutr..

[11] Islam M.T., Biswas S., Bagchi R., Khan M.R., Khalipha A.B.R., Rouf R., Uddin S.J., Shilpi J.A., Bardaweel S.K., Sabbah D.A. (2019). Ponicidin as a promising anticancer agent: Its biological and biopharmaceutical profile along with a molecular docking study. Biotechnol. Appl. Biochem..

[12] State Administration of Traditional Chinese Medicine (1999). Chinese Materia Medica.

[13] Zhang X., Xu Y., Fan M., Lv X., Long J., Yang R., Zhang R., Liu Z., Gu J., Wu P. (2024). Ponicidin-induced conformational changes of HSP90 regulates the MAPK pathway to relieve ulcerative colitis. J. Ethnopharmacol..

[14] Wang P., Jiang H., Yao J., He G., Tao T., Qin Z. (2025). The effect of ponicidin on CFA-induced chronic inflammatory pain and its mechanism based on network pharmacology and molecular docking. Front. Med..

[15] Li X., Li W., Zang C., Yan J., Cai M., Liu Z., Cai R., Gao Y., Qi Y. (2024). Hua-Shi-Bai-Du decoction inactivates NLRP3 inflammasome through inhibiting PDE4B in macrophages and ameliorates mouse acute lung injury. Phytomedicine.

[16] Hoss F., Rolfes V., Davanso M.R., Braga T.T., Franklin B.S. (2018). Detection of ASC Speck Formation by Flow Cytometry and Chemical Cross-linking, Methods. Mol. Biol..

[17] Jenster L., Ribeiro L.S., Franklin B.S., Bertheloot D., Clifton N.J. (2023). Measuring NLR Oligomerization II: Detection of ASC Speck Formation by Confocal Microscopy and Immunofluorescence. Methods in Molecular Biology.

[18] Victorelli S., Salmonowicz H., Chapman J., Martini H., Vizioli M.G., Riley J.S., Cloix C., Hall-Younger E., Machado Espindola-Netto J., Jurk D. (2023). Apoptotic stress causes mtDNA release during senescence and drives the SASP. Nature.

[19] Jakobs C., Bartok E., Kubarenko A., Bauernfeind F., Hornung V. (2013). Immunoblotting for active caspase-1, Methods. Mol. Biol..

[20] Martin W.J., Walton M., Harper J. (2009). Resident macrophages initiating and driving inflammation in a monosodium urate monohydrate crystal-induced murine peritoneal model of acute gout. Arthritis. Rheum..

[21] Rashidi M., Simpson D.S., Hempel A., Frank D., Petrie E., Vince A., Feltham R., Murphy J., Chatfield S.M., Salvesen G.S. (2019). The Pyroptotic Cell Death Effector Gasdermin D Is Activated by Gout-Associated Uric Acid Crystals but Is Dispensable for Cell Death and IL-1β Release. J. Immunol..

[22] Zhong C.S., Zeng B., Qiu J.H., Xu L.H., Zhong M.Y., Huang Y.T., Xu R., Liu S.Y., Zha Q.B., Hu B. (2022). Gout-associated monosodium urate crystal-induced necrosis is independent of NLRP3 activity but can be suppressed by combined inhibitors for multiple signaling pathways. Acta. Pharmacol. Sin..

[23] González-Cofrade L., Green J.P., de Las Heras B., Terencio M.C., Hortelano S., Brough D., Estévez-Braun A., Ferrándiz M.L., Cuadrado I. (2025). Dehydroisohispanolone alleviates NLRP3-driven inflammation in gout arthritis. Biochem. Pharmacol..

[24] Paik S., Kim J.K., Shin H.J., Park E.J., Kim I.S., Jo E.K. (2025). Updated insights into the molecular networks for NLRP3 inflammasome activation. Cell. Mol. Immunol..

[25] Lee A.H., Ledderose C., Li X., Slubowski C.J., Sueyoshi K., Staudenmaier L., Bao Y., Zhang J., Junger W.G. (2018). Adenosine Triphosphate Release is Required for Toll-Like Receptor-Induced Monocyte/Macrophage Activation, Inflammasome Signaling, Interleukin-1β Production, and the Host Immune Response to Infection. Crit. Care. Med..

[26] Malireddi R.K.S., Kanneganti T.D. (2022). Humans pIKK-up NLRP3 to skip NEK7. Trends. Immunol..

[27] Xue D., Ni F., Liu S., Yan H., Luo Z., Fu G., Wang Q., Ma J. (2025). Atomic mechanisms of full-length ASC-mediated inflammasome assembly. Nat. Commun..

[28] Ratsimandresy R.A., Dorfleutner A., Stehlik C. (2013). An Update on PYRIN Domain-Containing Pattern Recognition Receptors: From Immunity to Pathology. Front. Immunol..

[29] Shin H.J., Kim I.S., Kim J.K., Jo E.K. (2026). Molecular mechanisms of NLRP3 inflammasome activation. Exp. Mol. Med..

[30] Park Y.H., Batu E.D., Matthiasardottir B., Kim Y., Akkaya-Ulum Y.Z., Kastner D.L., Chae J.J. (2026). Cofilin-1 is a redox-sensitive guard of the NLRP3 inflammasome. Nat. Immunol..

[31] Baik S.H., Ramanujan V.K., Becker C., Fett S., Underhill D.M., Wolf A.J. (2023). Hexokinase dissociation from mitochondria promotes oligomerization of VDAC that facilitates NLRP3 inflammasome assembly and activation. Sci. Immunol..

[32] Zhong Z., Liang S., Sanchez-Lopez E., He F., Shalapour S., Lin X.J., Wong J., Ding S., Seki E., Schnabl B. (2018). New mitochondrial DNA synthesis enables NLRP3 inflammasome activation. Nature.

[33] Suomalainen A., Nunnari J. (2024). Mitochondria at the crossroads of health and disease. Cell.

[34] Ye L., Fu X., Li Q. (2025). Mitochondrial Quality Control in Health and Disease. MedComm.

