# Supplementary material for: Ponicidin Inhibits NLRP3 Inflammasome Activation via Alleviating Mitochondrial Damage in LPS-Primed Macrophages
Source: Antioxidants (Basel). 2026 Oct 14;15(10):1325. doi: 10.3390/antiox15101325 (PMC13701810; doi:10.3390/antiox15101325)
Supplement: Supplementary file 1 [file antioxidants-15-01325-s001.zip › Supplementary file.pdf]

Supplementary file

Supplementary Table S1. Primer sequences used in RT-qPCR analysis of IL-1β.

Gene		Primer sequences
IL-1β	F	5′ -GCA ACT GTT CCT GAA CTC AAC T-3′
	R	5′ -ACT TTT TGG GGT CCG TCA ACT-3′
GAPDH	F	5′ -GGT TGT CTC CTG CGA CTT CA-3′
	R	5′ -TGG TCC AGG GTT TCT TAC TCC-3′

F: forward primer; R: reverse primer

Supplementary Figure S1

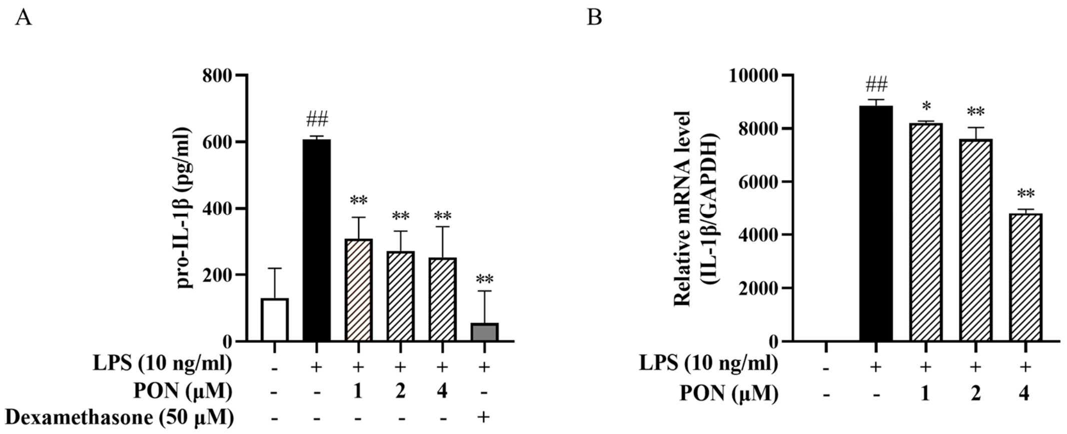

Figure S1. PON suppresses pro-IL-1β in LPS-stimulated RAW264.7 cells. (A) Effect of PON on pro-IL-1β of the cell lysates in the LPS-stimulated RAW264.7 cells. Cells were incubated with PON at indicated concentrations for 1 h, then exposed to LPS (10 ng/mL) for 24 h. The levels of intracellular pro-IL-1β in the cell lysates were detected using their ELISA kits. Dexamethasone was used as a positive control. (B) Effect of PON on the mRNA expression of IL-1β in LPS-stimulated RAW264.7 cells. Cells were pretreated with PON for 1 h, and then exposed to LPS (10 ng/mL) for 4 h. Total RNA was extracted, and the mRNA level was determined by RT-*q*PCR assays. Data were presented as the mean ± SD (n = 3). $^{\#\#}P < 0.01$ *vs*. negative control; $^{*}P < 0.05$ and $^{**}P < 0.01$ *vs*. LPS alone.

Supplementary Figure S2

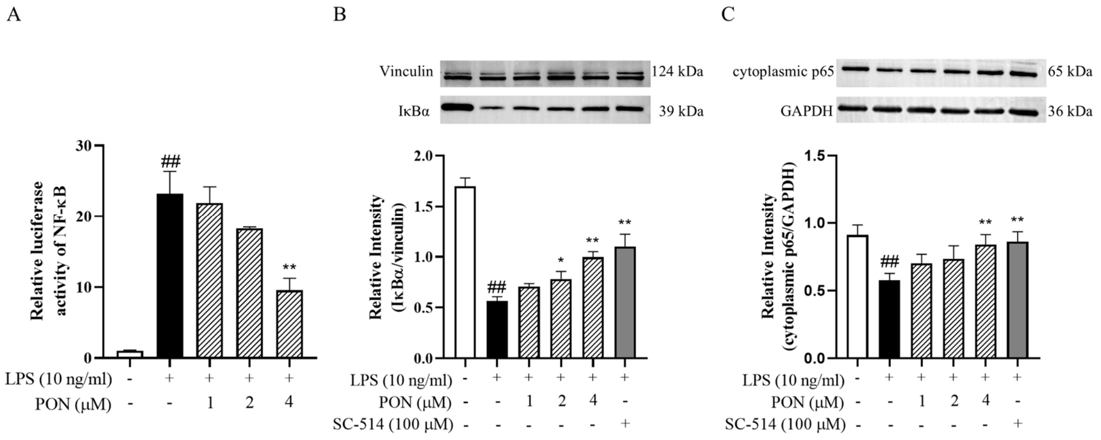

Figure S2. PON inhibits NF-κB pathway in LPS-stimulated RAW264.7 cells. (A) Effect of PON on the activity of NF-κB. The cells transfected with pNFκB-TA-luc luciferase reporter plasmid were pretreated with PON at the indicated concentrations for 1 h and then exposed to LPS (10 ng/mL) for 4 h. NF-κB activity was detected using luciferase assay system. (B-C) Effects of PON on LPS-induced degradation of IκBα and cytoplasmic p65. The cells were pretreated with PON at the indicated concentrations for 1 h and then stimulated with LPS (10 ng/mL) for 30 min. Cytoplasmic and total proteins were isolated and subjected to Western blot analysis. Data were presented as the mean ± SD (n = 3). $^{##}P < 0.01$ *vs*. negative control; $^{*}P < 0.05$ and $^{**}P < 0.01$ *vs.* LPS alone.
